# Supplementary material for: Divalent Cation Modulation of Ion Permeation in TMEM16 Proteins
Source: Int J Mol Sci. 2021 Feb 23;22(4):2209. doi: 10.3390/ijms22042209 (PMC7926781; doi:10.3390/ijms22042209)
Supplement: Supplementary file 1 [file ijms-22-02209-s001.pdf]

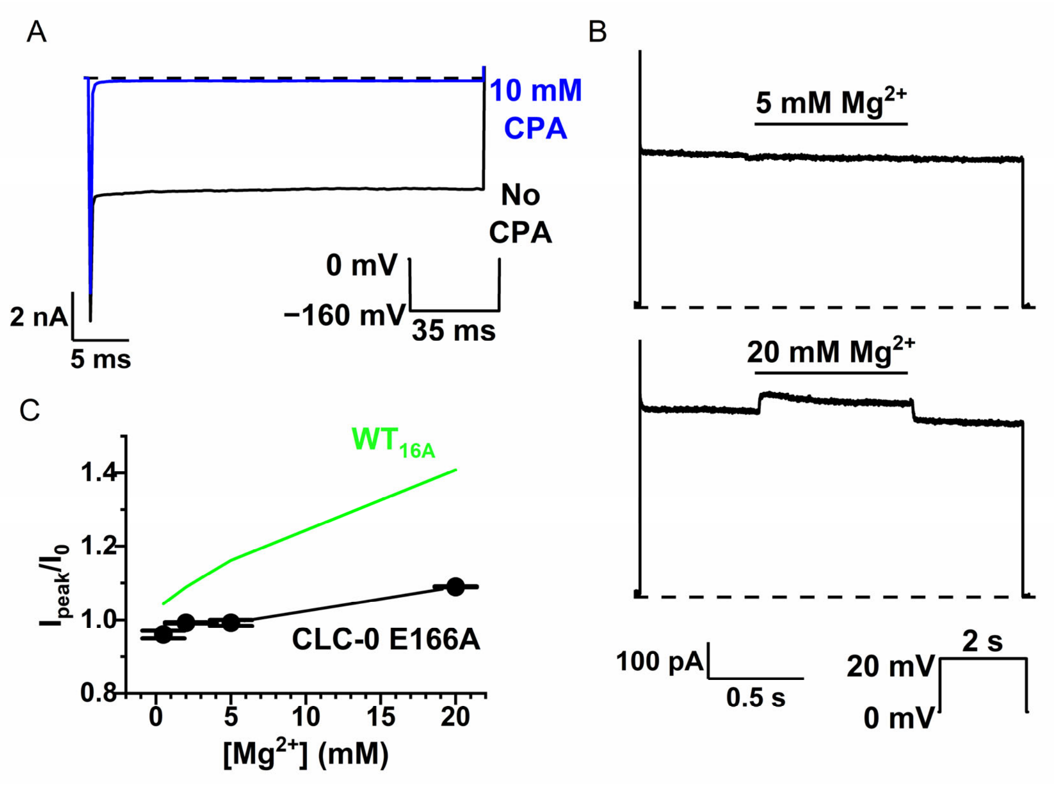

Figure S1. Potentiation of the current of E166A$_{CLC0}$ by intracellular Mg^{2+}. (A) Nearly complete current block of E166A$_{CLC0}$ by 10 mM CPA at -160 mV to ensure that the majority of the current in the recording patch was from the CLC-0 mutant. Horizontal dashed line indicates zero-current level. Inset shows the voltage protocol (from 0 mV to -160 mV) used to induced the current. **(B)** Representative traces showing the effect of 5 mM (upper panel) and 20 mM Mg^{2+} (lower panel) on the current of E166A$_{CLC0}$ at +20 mV. Inset shows the voltage protocol (0 mV to +20 mV) used for the experiments. Dashed lines are the zero-current level. **(C)** Dose-dependent Mg^{2+} potentiation of the E166A$_{CLC0}$ current (n = 3). The solid green line represents the Mg^{2+}-dependent potentiation of WT$_{16A}$.
